# A recombinant attenuated Mycobacterium tuberculosis-SIV combination vaccine is safe and immunogenic in immunocompromised, SIV-infected infant macaques

**DOI:** 10.1186/1742-4690-9-S2-O4

**Published:** 2012-09-13

**Authors:** K Jensen, UD Ranganathan, P Kozlowski, K Van Rompay, D Canfield, R Ravindran, I Khan, P Luciw, G Fennelly, M Larsen, K Abel

**Affiliations:** 1 Albert Einstein College of Medicine, New York, NY, USA; 2 LSUHSC, New Orleans, LA, USA; 3 UC Davis-CNPRC, Davis, CA, USA; 4 UC Davis - CNPRC, Davis, CA, USA; 5 UCD CCM, Davis, CA, USA; 6 UC Davis- CCM, Davis, CA, USA; 7 UNC Chapel Hill, Chapel Hill, NC, USA

## Background

HIV and Tuberculosis show high co-prevalence and cause high morbidity and mortality, especially in infants. There is no HIV vaccine, and the only licensed TB vaccine, BCG, can cause disseminated disease in HIV-infected infants. We propose to develop a pediatric combination HIV-TB vaccine hypothesizing that a highly attenuated strain of M. tuberculosis (AMtb) (i) would result in improved safety, but similar immunogenicity compared to BCG, (ii) could be modified to co-express HIV genes (rAMtb-HIV), and (iii) therefore induce HIV and Mtb specific immunity. Towards this goal, we tested distinct H37Rv Mtb mutants that differed in their attenuation for replication and immune evasion in infant rhesus macaques.

## Methods

Six infants that were SIV infected at birth, and 20 healthy infants received rAMtb-SIV orally at one week of age, and were followed for 3-6 months. Vaccine safety was assessed by clinical monitoring, histopathology, and Mtb detection using multiple culture methods. SIV and Mtb-specific T cell responses were measured by flow cytometry. Plasma, saliva and stool were tested for SIV and Mtb antibody responses.

## Results

Among three vaccine tested, the rAMtb-SIV strain mc6435 with deletions in panCD, LeuCD, and secA2 was determined to be safe in healthy and SIV-infected infant macaques. Lungs and other tissues were free of TB pathology, and viable mycobacilli could not be recovered from any tissues despite severe immune suppression by SIV.

Immunized animals showed increased dendritic cell activation, elicited polyfunctional SIV- and TB-specific CD4+ and CD8+ T cell responses in blood and tissues, and developed plasma IgG antibodies specific for Mtb and for SIV. The effectiveness of oral immunization was confirmed by detection of mucosal IgA responses to SIV in saliva and stool.

## Conclusion

A rAMtb-SIV vaccine is a safe alternative to BCG, is immunogenic in infants, and could be developed as a combination vaccine against pediatric HIV and Mtb infections.

